# *Announcement*: Release of National Association of State Public Health Veterinarians’ 2016 Compendium of Animal Rabies Prevention and Control

**DOI:** 10.15585/mmwr.mm6605a5

**Published:** 2017-02-10

The 2016 Compendium of Animal Rabies Prevention and Control was released in the March 1, 2016 issue of the Journal of the American Veterinary Medical Association (*1*). The Compendium’s national recommendations for the prevention and control of animal rabies are intended to serve as a basis for an effective rabies control program in the United States. These recommendations facilitate standardization of control procedures across jurisdictions and are reviewed annually and updated as necessary. This announcement of the recommendations facilitates their adoption by increasing awareness among public health agencies and practitioners and makes more readily available a link to statutes and regulations in certain jurisdictions that refer directly to the Compendium language published in *MMWR*.

The 2016 Compendium is an update and supersedes recommendations made in previous versions (*2*). Several modifications were made, including explicit encouragement of interdisciplinary approaches to rabies control, recommendations to collect and report additional data elements on rabid domestic animals to the national level, and updates to the list of marketed animal rabies vaccines.

The 2016 Compendium also includes important changes to the recommended management of dogs and cats exposed to rabies and a reduction of the recommended quarantine period for certain species. These recommendations are based on a combination of research indicating rapid and robust anamnestic response to booster doses of rabies vaccine, observational evidence of rabies incubation periods in exposed dogs and cats, and expert opinion. These particular recommendations are as follows: 1) dogs and cats that have never been vaccinated should either be euthanized immediately or vaccinated within 96 hours of the exposure and placed in strict quarantine for 4 months (a reduction from 6 months in previous recommendations); 2) dogs and cats that are overdue for a booster vaccination (and have appropriate documentation of prior rabies vaccination) should receive a booster vaccination within 96 hours of rabies exposure and be kept under owner observation for 45 days; and 3) dogs and cats that are overdue for booster vaccination (and do not have documentation of prior vaccination) may be treated as unvaccinated or undergo serologic monitoring to document an anamnestic response after receipt of a rabies booster vaccination.
